# Detection of Spinal Cord Multiple Sclerosis Lesions Using a 3D-PSIR Sequence at 1.5 T

**DOI:** 10.1007/s00062-023-01376-x

**Published:** 2024-01-30

**Authors:** Sönke Peters, Fernando Bueno Neves, Monika Huhndorf, Friederike Gärtner, Klarissa Stürner, Olav Jansen, Mona Salehi Ravesh

**Affiliations:** 1 Department of Radiology and Neuroradiology, University Hospital of Schleswig-Holstein, Campus Kiel, Arnold-Heller-Str. 3, 24105 Kiel, Germany; 2 Department of Neurology, University Hospital of Schleswig-Holstein, Campus Kiel, Kiel, Germany

**Keywords:** Multiple sclerosis, MRI, Spinal cord, STIR, T_2_-weighted

## Abstract

**Purpose:**

Multiple sclerosis (MS) is a prevalent autoimmune inflammatory disease. Besides cerebral manifestations, an affection of the spinal cord is typical; however, imaging of the spinal cord is difficult due to its anatomy. The aim of this study was to assess the diagnostic value of a 3D PSIR pulse sequencing at a 1.5 T magnetic field strength for both the cervical and thoracic spinal cord.

**Methods:**

Phase sensitive inversion recovery (PSIR), short tau inversion recovery (STIR) and T_2_-weighted (T_2_-w) images of the spinal cord of 50 patients were separately evaluated by three radiologists concerning the number and location of MS lesions. Furthermore, lesion to cord contrast ratios were determined for the cervical and thoracic spinal cord.

**Results:**

Of the lesions 54.81% were located in the cervical spinal cord, 42.26% in the thoracic spinal cord and 2.93% in the conus medullaris. The PSIR images showed a higher sensitivity for lesion detection in the cervical and thoracic spinal cord (77.10% and 72.61%, respectively) compared to the STIR images (58.63% and 59.10%, respectively) and the T_2_-w images (59.95% and 59.52%, respectively). The average lesion to cord contrast ratio was significantly higher in the PSIR images compared to the STIR images (*p* < 0.001) and the T_2_-w images (*p* < 0.001).

**Conclusion:**

Evaluation of the spinal cord with a 3D PSIR sequence at a magnetic field strength of 1.5 T is feasible with a high sensitivity for the detection of spinal MS lesions for the cervical as well as the thoracic segments. In combination with other pulse sequences it might become a valuable addition in an advanced imaging protocol.

## Introduction

Multiple sclerosis (MS) is a prevalent autoimmune inflammatory disease of the central nervous system (CNS) [1]. Magnetic resonance imaging (MRI) plays a crucial role in diagnosing MS and in disease and treatment monitoring [2]. Besides cerebral inflammation, MS causes demyelination in the spinal cord [3]. Imaging of the spinal cord of suspected MS patients is often essential to confirm the diagnosis of MS. The spinal cord is included in the dissemination of space in the McDonald diagnosis criteria for MS [4]. Furthermore, there can be a region of active contrast-enhancing lesions or new lesions in follow-up imaging to fulfil the criteria of dissemination in time [4]. In addition, spinal imaging can be helpful in discussing differential diagnoses of cerebral MRI findings as well as clarifying discrepancies between symptoms and cerebral MRI findings [3, 5, 6]. In patients with a verified diagnosis of MS, spinal cord imaging is helpful to estimate the prognosis as atrophy predicts a progressive disease [7, 8]. A higher spinal lesion load in the early disease stage is linked to a poor prognosis [9].

Imaging of the spinal cord is challenging, especially due to the relatively small crosssection and the extended configuration [10]. Requirements for imaging are therefore a relatively high spatial resolution with simultaneously high signal intensity and image contrast. Consequently, the recommended slice thickness for sagittal images is 3 mm or less [6]. Nevertheless, relatively small lesions in the spinal cord, as typical for MS, can be difficult to visualize and might lead to discrepancies of clinical and imaging findings [11]. Consequently, improving imaging quality is desired and the subject of various clinical studies [12–15]. At high magnetic field strengths of 3 T, phase sensitive inversion recovery (PSIR) images provided promising results in depicting more cervical MS lesions than other 2D or 3D noncontrast enhanced (CE) pulse sequences [13, 16]. For a magnetic field strength of 1.5 T, the 2D short tau inversion recovery (STIR) pulse sequence was superior in detecting cervical MS lesions compared with the 2D PSIR MRI technique [17].

This study aimed to evaluate the detectability of MS lesions in the spinal cord using 3D PSIR MRI at 1.5 T. Apart from the evaluation of the cervical spinal cord, as analyzed before with 3D PSIR at 3 T [16] and 2D PSIR at 1.5 T [17], we also examined the whole thoracic spinal cord with a 3D PSIR sequence and compared the detectability of MS lesions with STIR images and T_2_-w images.

## Material and Methods

The study was approved by the local ethics board of the medical faculty of the Christian-Albrechts-University Kiel (study number D624/20) and has been performed in accordance with the ethical standards laid down in the 1964 Declaration of Helsinki and its later amendments.

### Imaging

In this prospective study, between December 2020 and June 2021, in clinically indicated MRI examinations of the spinal cord for patients with known relapse remitting MS (RRMS), additional PSIR images were acquired. Patients were included in a consecutive manner. Exclusion criteria were other forms of MS. All images were obtained on a whole-body MRI system (1.5 T, MAGNETOM Aera, XQ gradients, Siemens Healthcare GmbH, Erlangen, Germany) with a maximum gradient strength of 45 mT/m and a maximum slew rate of 200 mT/m ms^−1^. The MR imaging system was operated by the syngo software (Versions E11C and E11E, Siemens Healthcare GmbH, Erlangen, Germany). The MR signal was received using a 20-element head coil, a 4-element neck coil, and a 32-element array coil placed on the upper chest (Siemens Healthcare GmbH). The MRI techniques used and their parameters are listed in Table 1.

**Table 1:** Protocol parameters of MRI pulse sequences used

MRI techniques	PSIR	STIR	T_2_-w	T_2_-w	CE T_1_
	Slice orientation				
Protocol parameters	Sagittal	Sagittal	Sagittal	Axial	Sagittal
TE (ms)	194	69	99	99	11
TR (ms)	4000	7430	4300	6830	570
TI (ms)	350	170	–	–	–
FA (°)	120	180	150	150	150
Slice thickness (mm)	1	3	3	3	3
Gap between the slices (mm)	–	0.3	0	6–7.5	0
Matrix	256 × 224	384 × 384	384 × 384	384 × 384	384 × 384
FOV (mm)	250 × 219	320 × 320	320 × 320	220 × 220	280 × 280

Protocol and sequence parameters used in the study. T_1_ weighted images were obtained after intravenous injection of a gadolinium-based contrast agent (0.1 mmol/kg BW gadobutrol).

*PSIR* phase sensitive inversion recovery, *STIR* short tau inversion recovery, *CE* contrast enhanced, *TR* time of repetition, *TE* time of echo, *TI* time of inversion, *FA* flip angle, *FOV* field of view

**Fig. 1:**
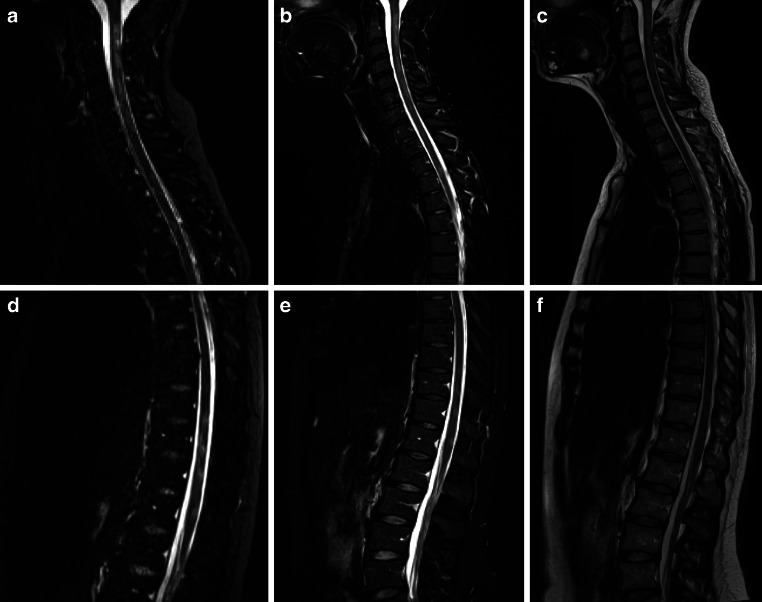
Exemplary acquired datasets with three different MRI techniques (PSIR, STIR and T_2_-w) in a 37-year-old female with a 15-year history of multiple sclerosis (MS) and an expanded disability status scale (EDSS) of 2. The spinal cord was examined with two stacks to cover the cervical and upper thoracic spinal cord (**a**–**c**) and the middle and lower thoracic spinal cord (**d**–**f**). MS lesions of the cervical and thoracic spinal cord are visible in the magnitude-3D-PSIR-MRI-images (**a** and **d**), the STIR images (**b** and **e**) and the T_2_ weighted images (**c** and **f**)

**Fig. 2:**
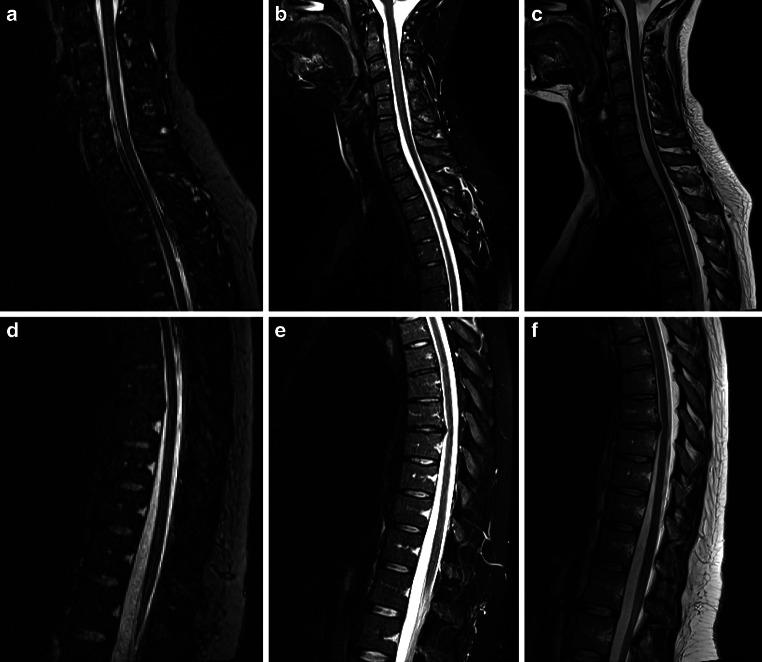
Example of a 30-year-old female patient with a 4-month history of multiple sclerosis (MS) but without spinal MS lesions. The expanded disability status scale (EDSS) was 1 and she had no disease-modifying treatment during the time of MRI. The imaging quality was rated good for the PSIR images (**a** and **d**) and for the STIR images (**b** and **f**). For the T_2_-w images (**c** and **f**) the imaging quality was rated good to very good

To evaluate the whole spinal cord, all contrasts were recorded with two stacks, the first covering the cervical and upper thoracic spine and the second covering the middle and lower thoracic spine (Figs. 1 and 2).

### Image Analysis

The images were analyzed by 3 radiologists with 3 (F.B.N), 10 (M.H.) and 12 (S.P.) years of experience in evaluating MR images. The radiologists with 10 and 12 years of experience were board certified neuroradiologists. The radiologist with 3 years of experience was a resident experienced in neuroradiology. They were blinded to clinical data and independently evaluated three imaging sets containing: 1) only the magnitude images of the 3D PSIR MRI technique; 2) the sagittal STIR images and the axial T_2_-w images and 3) the sagittal T_2_-w images combined with the axial T_2_-w images. After evaluating each imaging set the sagittal contrast enhanced (CE) T_1_-weighted (T_1_-w) images were evaluated for achieving additional information or lesions. The evaluation of each set of images was performed at least 6 weeks apart and the cases were presented in different order to avoid recognition.

First, the imaging quality was assessed separately for the cervical spinal cord, the thoracic spinal cord and the conus medullaris on a 5-point scale: 1) very good diagnostic imaging quality, 2) good diagnostic imaging quality with slight imaging artifacts but without impaired evaluation, 3) acceptable diagnostic imaging quality with slight impairment for evaluation, 4) poor diagnostic imaging quality with marked impairment for evaluation and 5) very poor diagnostic imaging quality that is not feasible to evaluate. Second, the reader counted the MS lesions in the cervical spinal cord, the thoracic spinal cord and the conus medullaris each, without a threshold concerning lesion size. Additionally, the lesions were separated by lateral, ventral/dorsal and central locations. Finally, the reader determined their certainty of the lesion count on a 3-point scale: 1) certain, 2) moderate and 3) uncertain, again separated by location as cervical, thoracic and conus medullaris.

To determine the ground truth, the images were re-evaluated for a consensus reading, including all pulse sequences, the clinical history and all results of the previous blinded reading available. Additionally, the normalized lesion to cord contrast ratio (CR lesion/cord) for the cervical spinal cord and the thoracic spinal cord was determined with the formula [18, 19]:

$$\mathrm{CR}=\frac{SI_{\text{Lesion}}-SI_{\text{Cord}}}{SI_{\text{Cord}}}$$

To measure the signal intensity (SI), regions of interest (ROI) were drawn manually in the most representative lesion of the cervical and thoracic spinal cord as well as in the neighboring unaffected spinal cord.

### Statistical Analysis

Normality (Gaussian distribution) was tested with the Shapiro-Wilk test. Due to skewness of the distribution the median and the range were reported for the patient age, the expanded disability status scale (EDSS) and the time elapsed between MS diagnosis and MRI examination. For the interrater agreement of lesion count the interclass correlation coefficient (ICC), two-way single agreement, was used. Values > 0.9 indicate an excellent agreement, 0.75–0.9 a good agreement, 0.5–0.74 a moderate agreement and < 0.5 a poor agreement [20]. Differences of CR lesion/cord were compared with the Wilcoxon rank sum test. A *p*-value < 0.05 was considered as statistically significant. The correlation of spinal cord lesions and the EDSS was determined with Spearman’s rank correlation coefficient, where 1.0 was considered as perfect, 0.8–0.99 as very strong, 0.6–0.79 as moderate, 0.3–0.59 as fair, 0.1–0.29 as poor and 0–0.09 as none [21].

The statistical analysis was performed with the software of the jamovi project (jamovi version 2.2.5; Retrieved from https://www.jamovi.org).

## Results

### Demographics

A total of 50 patients were included in the study, of whom 35 (70%) were female. The median patient age was 37 years (minimum 23 years and maximum 72 years) and the median time elapsed between MS diagnosis and MRI scan was 29.5 months (minimum 0 months maximum 249 months). The median EDSS of the patients was 2 (minimum 0 and maximum 6). With 46% the majority of patients had no MS medication during the MRI, followed by 30% of patients treated with ocrelizumab and 16% treated with dimethyl fumarate. Only 4% of the patients were treated with teriflunomide and 2% each were treated with interferon beta 1a and glatiramer acetate.

### Spinal Cord Lesions

**Fig. 3:**
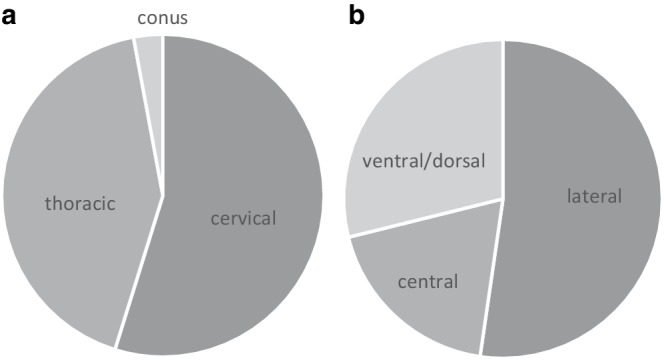
Overview of the anatomical and regional distribution of MS lesions in 50 study patients. **a** Of the MS lesions 54.81% were located in the cervical spinal cord, 42.26% in the thoracic spinal cord and 2.93% in the conus medullaris. **b** Of the MS-lesion 52.30% were located lateral in the spinal cord, 28.87% in the ventral or dorsal spinal cord and 18.83% were located centrally in the spinal cord

The 50 patients had 239 lesions in total, with a maximum of 22 lesions in 1 patient and 7 patients with no lesions (Fig. 2). Therefore, an average of 4.78 lesions per patient was detected. Of the 239 lesions, 131 (54.81%) were located in the cervical spinal cord, 101 (42.26%) in the thoracic spinal cord and 7 (2.93%) in the conus medullaris (Fig. 3a), 125 (52.30%) lesions were located laterally in the spinal cord, 69 (28.87%) in the ventral or dorsal spinal cord and 45 (18.83%) had a central location (Fig. 3b). Using Spearman’s rank correlation coefficient, the number of spinal cord lesions showed a fair correlation to the EDSS (r = 0.31; *p* = 0.03).

### Imaging Quality and Interrater Correlation

The mean imaging quality was rated as good to very good for all pulse sequences (Table 2). The interclass correlation coefficient (ICC) for the lesion count of the readers was 0.64 for PSIR, 0.82 for STIR and 0.74 for T_2_-w images.

**Table 2:** Evaluation of imaging quality in four different MRI techniques

MRI techniques with different slice orientation	Anatomical region		
	Cervical	Thoracic	Conus
PSIR sagittal	1.95 (0.81)	2.2 (0.77)	2.1 (1.10)
STIR sagittal	1.69 (0.82)	1.77 (0.65)	1.29 (0.03)
T_2_-w sagittal	1.47 (0.78)	1.57 (0.67)	1.39 (0.6)
T_2_-w axial	1.96 (0.94)	2.19 (0.66)	1.64 (0.82)
T_1_-w CE sagittal	1.33 (0.53)	1.19 (0.44)	1.4 (0.64)

Mean imaging quality and standard deviation (SD, bracketed) of the different MRI pulse sequences separated for the cervical spinal cord, the thoracic spinal cord and the conus medullaris. Where 1 means “very good diagnostic imaging quality” and 2 means “good diagnostic imaging quality with slight artefacts but without impaired evaluation”. *PSIR* Phase sensitive inversion recovery, *STIR* short tau inversion recovery, *T*_*2*_-*w* T_2_-weighted, *T*_*1*_-*w* T_1_-weighted, *CE* contrast enhanced

### Lesion Detection

With the sagittal and axial T_2_-w images 59.95% of the cervical lesions and 59.52% of the thoracic lesions were detected. With the sagittal STIR images and the axial T_2_-w images 58.63% of the cervical lesions and 59.10% of the thoracic lesions were detected. Using just the PSIR images 77.10% of the cervical lesions and 72.61% of the thoracic lesions were detected; however, with the PSIR images considerably more false positive lesions were counted, on average 43.67 per reader, compared to the T_2_-w images, on average 17.67 per reader, and compared to the STIR images, on average 13 per reader. The certainty of lesion count was certain to moderate in all groups, for details see Table 3. There was no case in which the CE T_1_-w images revealed additional information.

**Table 3:** Evaluation of certainty of lesion count using three different noncontrast enhanced MRI techniques

Anatomical regions	MRI techniques		
	PSIR	STIR	T_2_-w
Cervical	1.36 (0.62)	1.39 (0.62)	1.36 (0.61)
Thoracic	1.44 (0.65)	1.37 (0.59)	1.54 (0.64)
Conus	1.1 (0.36)	1.06 (0.30)	1.13 (0.44)
Total	1.34 (0.60)	1.27 (0.54)	1.34 (0.60)

Average certainty of lesion count and standard deviation (SD, bracketed), separated by location as cervical spinal cord, thoracic spinal cord and conus medullaris and in total. The certainty was rated on a 3-point scale: 1) certain; 2) moderate; 3) uncertain

### Lesion to Cord Contrast Ratio

**Fig. 4:**
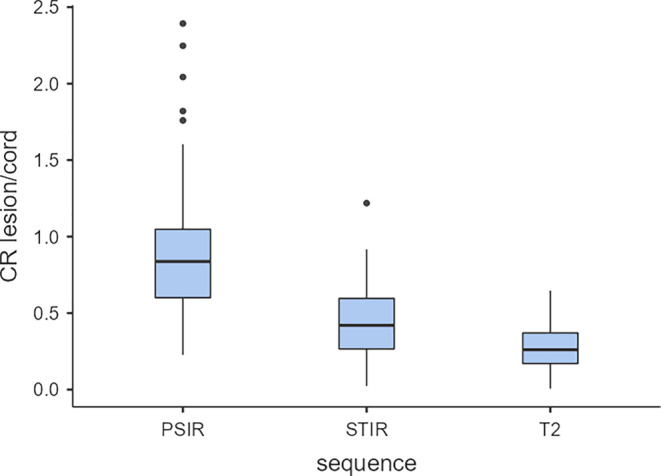
Box and whisker plot of signal intensity differences of MS lesions to neighbouring healthy spinal cord tissue. The ratio between the signal intensities of an MS lesion and adjacent healthy spinal cord tissue is expressed as the lesion to spinal cord contrast ratio (CR lesion/spinal cord). The bars are marking the median CR lesion/spinal cord, the boxes are representing the upper and lower quartil, the whiskers including values within the 1.5 fold range of the interquartile range and the dots are outlier. *CR* contrast ratio, *PSIR* phase sensitive inversion recovery, *STIR* short tau inversion recovery

**Fig. 5:**
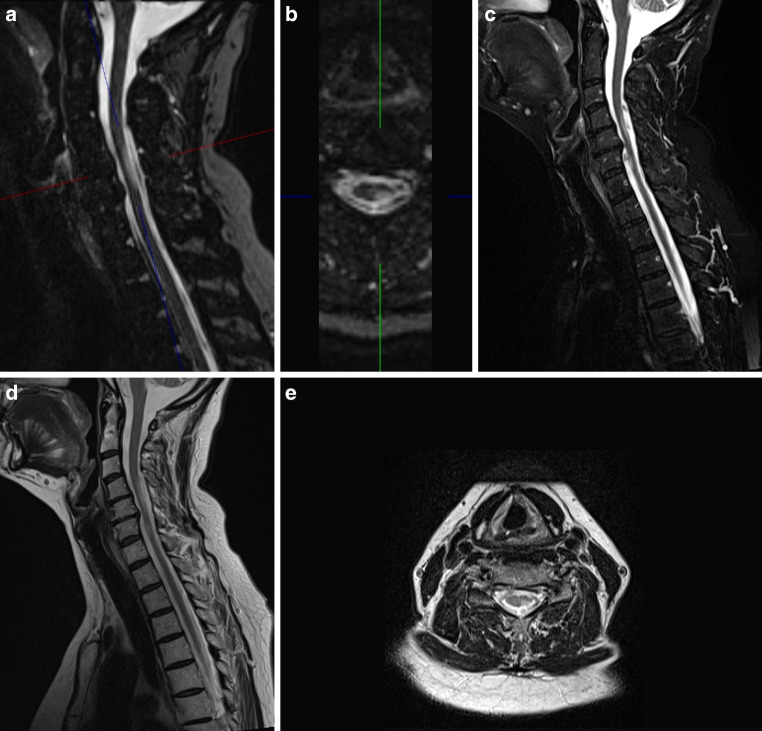
Example of a cervical MS lesion visible in the sagittal PSIR image (**a**) and the axial reconstructed PSIR image (**b**) as well as in the sagittal STIR image (**c**) and in the sagittal and axial T_2_-w images (**d** and **e**). For this lesion the lesion to cord contrast ratio was 1.4 in the PSIR image, 0.71 in the STIR image and 0.54 in the T_2_-w image

The average CR of lesion/cord was highest in the PSIR images (cervical 0.95 ± 0.40; thoracic 0.88 ± 0.50; total 0.92 ± 0.45), followed by the STIR images (cervical 0.44 ± 0.21; thoracic 0.44 ± 0.25; total 0.44 SD 0.23) and the lowest in the T_2_-w images (cervical 0.29 ± 0.15; thoracic 0.26 ± 0.15; total 0.28 ± 0.15) (Figs. 4 and 5).

Using the Wilcoxon rank sum test the differences between the PSIR-CRs, the STIR-CRs and the T_2_-w CRs were significant for the cervical spinal cord, the thoracic spinal cord and both locations taken together (*p* < 0.001 each). Similarly, the STIR-CRs were significantly higher than the T_2_-w CRs for all locations (*p* < 0.001).

## Discussion

This study shows that the evaluation of the whole spinal cord with the 3D PSIR MRI technique is feasible with a high sensitivity for the detection of spinal MS lesions at a magnetic field strength of 1.5 T. Compared to two other non-CE pulse sequences, it has a higher lesion to cord contrast and improves the sensitivity for lesion detection.

Two types of images (phase corrected real images and magnitude) can be reconstructed from the same acquired dataset using the PSIR MRI technique. For 2D PSIR images at 1.5 T, a higher rate of lesion detection was described for the magnitude images compared to the phase corrected real images [17]. We therefore choose to evaluate the magnitude images of the PSIR sequence concerning lesion detection and CR lesion/cord.

In contrast to 2D PSIR images, which were inferior in lesion detection in the cervical spinal cord compared to STIR images at 1.5 T [17], in our study PSIR images showed a higher sensitivity compared to STIR images and T_2_-w images. A possible explanation is the higher spatial resolution of the 3D images compared to the 2D images. To compensate this limitation the 2D images were recorded in two different planes. Nevertheless, the 2D sequences are the clinical standard [6] and therefore the benchmark.

A superiority of 3D PSIR images in evaluating the cervical spinal cord at 3 T has already been described [16, 19, 22]; however, to our knowledge this is the first study evaluating a 3D PSIR at a magnetic field strength of 1.5 T. In contrast to the findings at 3 T, we could not confirm a rising interrater correlation using PSIR images. In our study, the interrater correlation was the highest for the STIR images. Besides the different magnetic field strengths, a possible reason for this might be a different lesion threshold. We also counted small and somewhat blurry lesions, whereas Fechner et al. stated “not to count lesions in areas too full of artifacts and to report only evident and well-delimitated lesions” [16]. Together with the lower magnetic field strength, this is also a possible explanation for the false positive lesions counted in our study, a phenomenon not reported by the French working group [16, 19]. The fact that we also tried to count small lesions without a minor size limit and blurry lesions might also explain the higher false positive rate in the 3D images in our study compared to the 2D images; however, we had a higher sensitivity using the 3D PSIR images. Even though the McDonald criteria are intended for MS diagnosis and not for follow-up evaluations, the herein defined lower lesion size of 3 mm [4] will probably decrease false positive results and therefore increase specificity; however, in clinical routine we also observe smaller MS lesions in the spinal cord [23]. Considering the relatively small diameter of the spinal cord [10], and the close relation of functionally important structures as well as for considerations of differential diagnoses in patients without the diagnosis of MS yet, these small lesions are also clinically relevant. Furthermore, a too restrictive definition of lesion size would lower the sensitivity of the examination. A possible solution might be a combination of a 3D and a 2D pulse sequence in an advanced examination protocol of the spinal cord in MS patients. The MAGNIMS-CMSC-NAIMS working group recommended using at least two sagittal images for MS diagnosis [6]. In the aforementioned consensus recommendations from 2021 [6], PSIR images are mentioned as optional due to a lack of data and clinical experience. According to our results, PSIR and STIR seem to be the best partners. This is in concordance with the literature, where STIR images are described as superior to T_2_-w images at both 1.5 T and 3 T [24–27]. Nonetheless, this was not the goal of this study and could be addressed by future research.

There are less frequent clinical research studies concerning the using of PSIR MRI technique in the thoracic spinal cord. Regarding 2D pulse sequences at a magnetic field strength of 3 T, PSIR has a lower sensitivity than STIR and T_2_-w images [13]. With a 3D SPIR at 3 T lesion detection in the upper thoracic segments seems to be reliable [19]; however, the aforementioned study [19] did not cover the whole spinal cord, on average only down to the 8th thoracic segment. Our results showed that even at a lower magnetic field strength of 1.5 T, a 3D PSIR rises the sensitivity for lesion detection in the thoracic spinal cord.

Besides the higher spatial resolution in 3D pulse sequences, higher image contrast between the spinal cord and the MS lesions are discussed as possible reasons for the superior lesion detection and lesion counts in 2D [13] and 3D PSIR images at 3 T [16, 19]. We can confirm these findings for a magnetic field strength of 1.5 T, as we found significantly higher CR lesion/cord in the 3D PSIR images compared to the 2D STIR and the 2D T_2_-w images (Figs. 4 and 5).

To reduce the total examination time, a relatively wide gap was employed in the axial T_2_-w images in this study. This implies a limitation for lesion detection. Still, axial imaging is mentioned as optional in international imaging guidelines [6]. Even though it can be regarded as a dispensable addition to sagittal images, an increased slice thickness might have been more favorable instead.

Even though spinal cord lesions are of predictive value in clinically isolated syndrome (CIS) and radiologically isolated syndrome (RIS) patients developing MS [28, 29], the spinal lesion count only correlates moderately with the EDSS in our study and poorly to moderately in other studies [19, 22]. Nevertheless, a high sensitivity for lesion detection is important concerning differential diagnostic considerations as well as for the assessment of disease activity and the monitoring of effectiveness of the disease-modifying treatment. If there is a general improvement in lesion detection using 3 T instead of 1.5 T magnetic field strengths remains unclear [6, 30]. For PSIR pulse sequence a benefit for lesion detection in the cervical spinal cord is described, as discussed above. We could confirm a rising sensitivity using 3D PSIR in routinely widely available MRI systems with a magnetic field strength of 1.5 T. A direct comparison of 3D PSIR images at 1.5 T and 3 T in future studies would be of interest to provide optimal diagnostic imaging for MS patients.

## Conclusion

The use of 3D PSIR images improves the sensitivity of lesion detection in the cervical and thoracic spinal cord at a magnetic field strength of 1.5 T. In combination with other non-CE pulse sequences the lower specificity might be compensated and therefore 3D PSIR images might become a valuable complimentary non-CE MRI technique in an advanced imaging protocol for MS patients.

## Abbreviations

CE: Contrast-enhanced
CIS: Clinically isolated syndrome
CNS: Central nervous system
CR lesion/cord: Lesion:cord contrast ratio
EDSS: Expanded disability status scale
FA: Flip angle
FOV: Field of view
ICC: Interclass correlation coefficient
MRI: Magnetic resonance imaging
MS: Multiple sclerosis
PSIR: Phase sensitive inversion recovery
RIS: Radiologically isolated syndrome
ROI: Region of interest
RRMS: Relapse remitting MS
SD: Standard deviation
SI: Signal intensity
STIR: Short tau inversion recovery
T_1_-w: T_1_-weighted
T_2_-w: T_2_-weighted
TE: Time of echo
TI: Time of inversion
TR: Time of repetition
